# Time-lapse imaging of CD63 dynamics at the HIV-1 virological synapse by using agar pads

**DOI:** 10.17912/micropub.biology.000648

**Published:** 2022-10-07

**Authors:** Daniel Ivanusic, Kazimierz Madela, Norbert Bannert, Joachim Denner

**Affiliations:** 1 Sexually transmitted bacterial pathogens and HIV (FG18), Robert Koch-Institute, Nordufer 20, 13353 Berlin, Germany.; 2 Special Light and Electron Microscopy (ZBS4), Robert Koch-Institute, Nordufer 20, 13353 Berlin, Germany.; 3 Institute of Virology, Department of Veterinary Medicine, Free University Berlin, 14163 Berlin, Germany.

## Abstract

Time-lapse imaging provides an uninterrupted observation method that can lead to understanding protein dynamics. We previously developed a technique based on thin agar pads to keep the cells in focus during confocal laser scanning microscope imaging. Using this method, time-lapse imaging was employed to monitor CD63 fused to mCherry at the virological synapse (VS) during viral cluster transfer to acceptor cells of the human immunodeficiency virus 1 (HIV-1).

**Figure 1. Time-lapse monitoring of CD63-mCherry and CD63 C145A,C146A -mCherry protein dynamics at the virological synapse.**
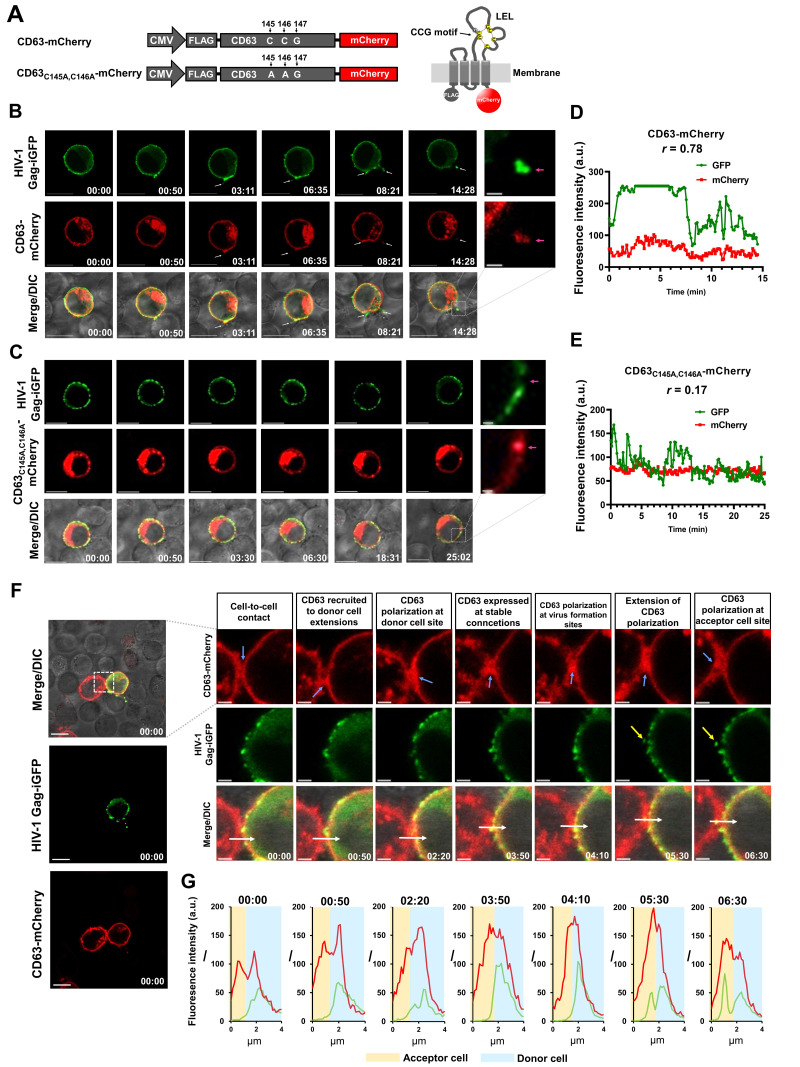
( **A** ) Schematic representation (not to scale) of the constructs expressing the CD63-mCherry fusion protein, numbers in the cartoon refer to the amino acid sequence. LEL = large extracellular loop, C = cysteine, G = glycine. Disulfide bridges are displayed as broken lines, constructs were additionally fused N-terminally to a FLAG-tag. ( **B** ) CD63-mCherry or ( **C** ) CD63 _C145A,C146A_ -mCherry protein dynamics at the virological synapse. Confocal microscopy images of cell-cell contacts (virological synapse, VS) between Jurkat cells expressing HIV-1 _JR-FL_ Gag-iGFP (green) and CD63-mCherry or with mutation LEL variant (red) were taken at indicated time points. White arrows depict transfer of viral Gag-iGFP clusters. Fluorescence intensity ( *I* ) profiles of detected signals emitted by the fluorescence proteins GFP (green line) and mCherry (red line) ( **D** ) for CD63-mCherry or ( **E** ) CD63 _C145A, C146A_ -mCherry during live-cell imaging are displayed. ( **F** ) Confocal microscopy images of Jurkat cells expressing CD63-mCherry (red) and one donor cell expressing HIV-1 _JR-FL_ Gag-iGFP (green). White arrow indicates the region for which the fluorescence intensity ( *I* ) was measured, the yellow arrow points to site where viral clusters are transferred, pale blue arrow depicts the situation described in above images. ( **G** ) The *I* values for GFP (green line) and mCherry (red line) signals are displayed as a function of the distance. For each graph the time point is given in mm:ss and correspond to the confocal images above. All scale bars are 10 µm, except in B and C cutouts, where they are 1 µm and in F cutouts they are 2 µm. Time is given in mm:ss.

## Description

Time-lapse microscopy for monitoring of fluorescently tagged proteins provides an undoubtedly important tool to understand transport, localization, and fate of proteins in living cells. Serial images are taken at consecutive time points to follow the cell dynamics. Using fixed cells can only provide a “snapshot” of what is occurring in the cells. It was challenging to keep cells in focus when suspension cells are observed. The use of agar pads as described previously (Ivanusic et al., 2017) is a technique forcing suspension cells in a particular location using an agar pad. This technique was used for monitoring cell-to-cell transfer of human immunodeficiency virus type 1 (HIV-1) over time, allowing observations of transient events that may have been missed when fixed cells are used. Transmission of virus particles through cell-to-cell contacts between productively infected and target CD4 ^+^ T-cells occurs after the formation of a cell-to-cell membrane site (Piguet and Sattentau, 2004). It was shown that CD63 and other tetraspanins regulate the cell–cell transmission of HIV-1 particles (Krementsov et al., 2009). This direct cell-to-cell transfer via a virological synapse (VS) without release and *de novo* infection of the virus is considered one of the strategies on how HIV-1 evades the immune system. This route of viral spread is far more efficient than infection with freely floating viral particles and allows HIV-1 to evade antibody neutralization (Chen et al., 2007). Although HIV-1 transfer through VS was already assessed by live cell imaging (Hubner et al., 2009), a study using the protein CD63, which is relevant for the VS, has not been reported so far. In our previous experiments (Ivanusic et al., 2021a) we were able to track the VS, but two interesting questions were raised. First, how specific is the expressed CD63-mCherry recruited to regions of HIV-1 _JRFL_ Gag-iGFP clustering? Second, how is the movement of cell extensions and the expression of CD63-mCherry organized at the VS? To answer these two questions and most importantly to confirm previous observations by an uninterrupted technique, we acquired time-lapse images using living Jurkat cells after applying the same transfection and handling techniques (Ivanusic et al., 2017, Ivanusic et al., 2021a). We characterized the dynamic formation of a VS using CD63-mCherry and a R5 tropic HIV-1 _JRFL_ Gag-iGFP molecular clone (a viral strain containing the JRFL envelope sequence). This clone contained the green fluorescence protein (GFP) flanked by a protease cleavage site inserted in the Gag protein (Hubner et al., 2007). In order to express CD63 variants, the CD63 sequences were fused N-terminally with a FLAG-tag and C-terminally with the red fluorescence protein (mCherry). Only VS were visualized that met the morphological features of stable cell-cell contacts enriched with the fluorescent virus for over 10 min. (Wang et al., 2017, Law et al., 2016). CD63, like other tetraspanins contains four transmembrane domains, a small and a large extracellular loop (SEL and LEL). The LEL contains a CCG motif that is highly conserved among the tetraspanin superfamily (DeSalle et al., 2010, Boucheix and Rubinstein, 2001, Stipp et al., 2003). Therefore, we used Jurkat cells transfected with the expression construct pCMV-CD63-mCherry containing cysteine-to-arginine (C-->A) mutations within the CCG motif of the LEL of CD63 as control ( **Fig. 1A** ). This mutation in CD63 was shown to abrogate recruiting CD63 to the VS (Ivanusic et al., 2021a). In order to monitor the dynamics and distribution of CD63-mCherry at the virological synapse of transfected Jurkat cells, they were monitored at different time points by confocal laser scanning microscopy. For this, the Jurkat cells were transfected by electroporation with the vectors pCMV-CD63-mCherry or the CD63LEL variant with the introduced mutations C145A,C146A and the molecular clone HIV-1 _JRFL_ Gag-iGFP. Non-transfected Jurkat cells were added 24 hours after transfection and the contacts between cells were monitored. After first contacts of the donor and acceptor cells ( **Fig. 1B** ) (time point 00:00), imaging of the VS revealed very dynamic movements and a rapid polarization of signals emitted by CD63-mCherry and polarized iGFP-Gag proteins at the site of a cell contact ( **Fig. 1B** ) (time point 03:11). Notably, accumulation of iGFP-Gag proteins together with CD63 on large areas of the surface of the acceptor cell implied an extension of the VS ( **Fig. 1B** ) (time point 08:21). After extending the VS, the acceptor cell contained only a fraction of the iGFP-Gag on the cell surface ( **Fig. 1B** ) (time point 14:28). In contrast to the wild-type CD63-mCherry, CD63-mCherry with the introduced cysteine point mutations showed an abrogation of the polarization of iGFP-Gag at the VS ( **Fig. 1C** ). Between the initial contact and attaching viral clusters on the acceptor cell, it took 8:21 min. The contact between donor and acceptor cell was still stable after 14:28 min. The fluorescence intensity of iGFP-Gag increases after initial contact with CD63-mCherry ( **Fig. 1B** ) (time point 00:50) until the iGFP-Gag cluster is transferred to the donor cell ( **Fig. 1B** ) (time point 14:28). In contrast, when CD63 _C145A,C146A_ -mCherry was expressed, the fluorescence intensity of iGFP-Gag is randomly polarized over all time points and showed no stable cell-cell contact and no formation of a VS ( **Fig. 1C** ). Furthermore, CD63 _C145A,C146A_ -mCherry does not colocalize with HIV-1 _JRFL_ iGFP-Gag surface clusters ( **Fig. 1C** , right panel). In contrast, CD63-mCherry is co-localized with HIV-1 _JRFL_ iGFP-Gag surface clusters even when they are transferred to the acceptor cell ( **Fig. 1B** , right panel). The fluorescence intensities for mCherry and GFP increased at the VS when the wildtype CD63 was expressed, but for mutated CD63 we observed a randomized increase together with GFP signals ( **Fig. 1D, 1E** ). The Pearson correlation coefficient (PCC) for the GFP signal derived from HIV-1 clusters when expressing CD63-mCherry is *r* = 0.78 and means a strong linear correlation between CD63-mCherry and GFP signals. In contrast, when CD63 _C145A,C146A_ -mCherry is expressed the PCC is *r* = 0.17 that would mean that there is a very weak linear correlation. In total we have observed in our preparation expressing CD63-mCherry seven VS formations in seven cell-cell contacts (7/7) showing similar situations but in contrast for cells in cell-cell contacts expressing CD63 _C145A,C146A_ -mCherry we have observed one VS formation out from nine (1/9) without recruitment of CD63 _C145A,C146A_ -mCherry to the VS. Next, we examined the organization of CD63 in T-cells during formation of a VS ( **Fig. 1F** ). We observed that CD63-mCherry, at first cell-cell contact, is polarized at the donor cell site ( **Fig. 1F** ) (time point 00:00). After establishing a cell-cell connection formed presumably by cell extensions, which formed gates through which HIV-1 _JRFL_ iGFP-Gag formations are transferred to the acceptor cell, CD63-mCherry was predominantly localized at these sites ( **Fig. 1F)** (time point 02:20). At the synaptic cleft the transferred HIV-1 _JRFL_ iGFP-Gag cluster is completely surrounded by CD63-mCherry ( **Fig. 1F** ) (time point 05:30). The intensities of the fluorescence peaks for CD63-mCherry showed that it is localized predominantly at the donor cell site. However, after transfer of HIV-1 _JRFL_ Gag-iGFP clusters mediated by connective gates between donor and acceptor cells, CD63-mCherry is predominantly detected at acceptor cell site ( **Fig. 1G** ). This data shows that the use of an agar pad is advantageous for the investigation of transient events of protein dynamics such as shown for CD63-mCherry at the VS.

## Methods

**Plasmids, Transfection and Cell Culture**

Jurkat cells were maintained and transfected as described (Ivanusic et al., 2021a). All used plasmids were described previously (Ivanusic et al., 2021a, Ivanusic et al., 2021b). Co-transfected cells with the molecular clone HIV-1 _JRFL_ Gag-iGFP and pCMV-CD63-mCherry or pCMV-CD63 _C145A,C146A_ -mCherry are transfected with a plasmid DNA ratio of 1:1.

**Confocal Laser Scanning Microscopy (cLSM)**

All images were acquired using a confocal laser scanning inverted microscope device, Zeiss LSM 780 (Carl Zeiss, Oberkochen, Germany), with a 63x objective with oil immersion. Fluorescence signals were detected with settings for 488 nm (2.6 % laser power) and 594 nm (4.0 % laser power) dyes, time series function was set at 10 s intervals. For live cell visualization, sedimented Jurkat cells 24 h after transfection co-expressing HIV-1 _JR-FL_ Gag-iGFP, CD63-mCherry or CD63 _C145A,C146A_ -mCherry were transferred in an IBIDI µ-Dish, 35 mm high (IBIDI, Martinsried, Germany) as previously published (Ivanusic et al., 2017). Uninfected Jurkat cells were added before agar pad was applied and then the IBIDI µ-Dish was placed in a prewarmed microscope with an environmental chamber at ambient temperature of 37 °C. Serial time-lapse images at indicated time points were obtained by automatic acquisition in order to follow up with fluorescently expressed fused proteins at cell-cell contacts.
